# Data-driven prediction of continuous renal replacement therapy survival

**DOI:** 10.21203/rs.3.rs-3487939/v1

**Published:** 2023-11-14

**Authors:** Davina Zamanzadeh, Jeffrey Feng, Panayiotis Petousis, Arvind Vepa, Majid Sarrafzadeh, S Ananth Karumanchi, Alex A. T. Bui, Ira Kurtz

**Affiliations:** 1 Department of Computer Science, University of California, Los Angeles, Log Angeles, 90095, California, United States.; 2 Medical & Imaging Informatics Group, University of California, Los Angeles, Los Angeles, 90095, California, United States.; 3 Clinical and Translation Science Institute, University of California, Los Angeles, Los Angeles, 90095, California, United States.; 4 Department of Medicine, David Geffen School of Medicine, University of California, Los Angeles, Los Angeles, 90095-1689, California, United States.; 5 Department of Medicine, Cedars-Sinai Medical Center, Los Angeles, 90048, California United States.

## Abstract

Continuous renal replacement therapy (CRRT) is a form of dialysis prescribed to severely ill patients who cannot tolerate regular hemodialysis. However, as the patients are typically very ill to begin with, there is always uncertainty as to whether they will survive during or after CRRT treatment. Because of outcome uncertainty, a large percentage of patients treated with CRRT do not survive, utilizing scarce resources and raising false hope in patients and their families. To address these issues, we present a machine-learning-based algorithm to predict if patients will survive after being treated with CRRT. We use information extracted from electronic health records from patients who were placed on CRRT at multiple institutions to train a model that predicts CRRT survival outcome; on a held-out test set, the model achieved an area under the receiver operating curve of 0.929 (CI=0.917–0.942). Feature importance, error, and subgroup analyses identified consistently, mean corpuscular volume as a driving feature for model predictions. Overall, we demonstrate the potential for predictive machine-learning models to assist clinicians in alleviating the uncertainty of CRRT patient survival outcomes, with opportunities for future improvement through further data collection and advanced modeling.

## 1 Introduction

Renal replacement therapy (RRT) encompasses a range of treatments that replace some of the capabilities of inadequately functioning kidneys [1]. Certain patients, typically because of hemodynamic compromise, are unable to tolerate the most common form of RRT, hemodialysis, and are instead considered for continuous renal replacement therapy (CRRT), which provides gentler treatment over a more prolonged period of time [2, 3]. Despite several decades of use, there remains no widely-adopted consensus on clinical guidelines physicians should use to decide whether to initiate CRRT that will result in a good outcome [4–7]. The decision whether to put a patient on CRRT largely depends on the physician’s assessment of the patient’s medical history, vital signs, labs, and medications [8]. Unfortunately, it is estimated that approximately 50% of adults who are placed on CRRT do not survive [5, 9–12], and as such, treatment with CRRT is often futile for both the patient and their families. Adding to this issue is the approach in tertiary and quaternary care centers to “pull out all the stops”, resulting in the treatment of patients with CRRT as a last hope to survive [13]. Mitigating this uncertainty is important not only to ensure that CRRT is recommended to patients who will benefit from the treatment but also to discern those who will not, as it is a resource-intensive intervention (time, personnel, equipment, cost) and when used inappropriately is not in concert with the ideal approach to managing patients. A predictive tool to inform this clinical decision-making task can improve the number of positive patient outcomes, help optimize resource allocation, and provide support for clinicians to explain their decisions to the patients and their families.

We introduce a machine learning model on a target outcome of whether a patient should start CRRT. Unlike existing models that predict in-hospital mortality once CRRT *has started* [9, 10, 14–16], our model informs the clinician as to whether CRRT *should be initiated* in the first place. We provide a unique and in-depth analysis of our predictive model and utilize a large, longitudinal dataset of patients who were placed on CRRT at University of California, Los Angeles (UCLA) and Cedars-Sinai Medical Center quaternary care hospitals in Los Angeles. We highlight the needed clinical parameters in several patient subgroups that should be monitored and evaluated prior to CRRT initiation to inform the key clinical decision as to whether a patient will survive CRRT treatment or not.

## 2 Results

### 2.1 Machine learning for predicting patient outcomes on CRRT

**Fig. 1:**
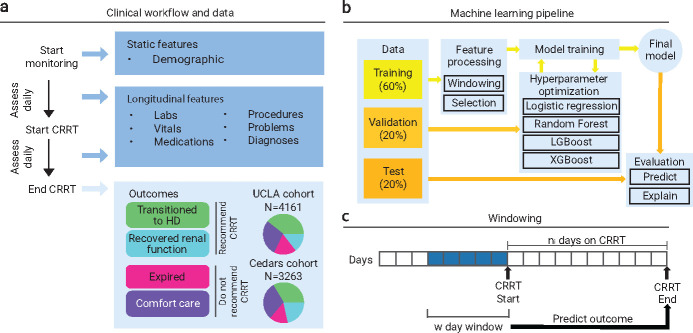
Overview of study design and machine learning framework. a) Data and outcome labels. Two cohorts of data consisting of patients put on CRRT were collected from the UCLA and Cedars Sinai hospital systems. Static features were collected once at the outset of initiating CRRT. Longitudinal features were collected multiple times, before and after patients started CRRT. Patients who recover renal function or transition to hemodialysis experience a positive outcome, while patients who transition to hospice or expire experience a negative outcome. b) Schematic of the machine learning pipeline. Training, tuning, and validation were performed on a 60/20 split of the dataset, with the remaining 20% as a holdout test set. External testing is performed on any unseen cohort. c) Schematic of windowing of longitudinal features. Features were aggregated over a *w* day window prior to the first day of CRRT. The features are used to predict the final outcome, regardless of the number of days on CRRT.

We collected a dataset to develop a machine learning model to predict if a patient will survive after being placed on CRRT, consisting of three cohorts across two hospital systems for a total of *N* = 12,149 patients. The UCLA: CRRT (*N* = 4,161) and Cedars: CRRT (*N* = 3,263) cohorts contain adult patients treated at the respective hospitals who were all placed on CRRT. The UCLA: Control cohort (*N* = 4,725) contains adult patients treated at UCLA but were not placed on CRRT, matched to individuals in the UCLA: CRRT cohort (Methods 4.1). We unified four known outcomes (described in Methods 4.1) to construct the binary outcome variable of whether a patient should be placed on CRRT (Figure 1a), resulting in relatively balanced distributions for both cohorts (UCLA: *N* = 2,241 (53.9%) should be placed on CRRT; Cedars Sinai: *N* = 1,801 (55.2%) should be placed on CRRT).

We associate each sample (patient-treatment pair) with electronic health record (EHR) data that we process into various data tables (Figure 1a; Methods 4.1). The features for modelling are collected from data within a predefined window of days prior to starting CRRT to accurately represent the clinical question of whether a patient will benefit from CRRT (Figure 1c). After data preprocessing (Methods 4.2), we train, tune, and test multiple predictive models (Figure 1b; Methods 4.3.2). The train and validation splits consist of patients who were on CRRT for a maximum of seven days, which captures the majority of the patient population (UCLA: *N* = 2,435 (58.5%); Cedars Sinai: *N* = 2,069 (63.4%)). However, when applying the model to potential CRRT patients, it would not be possible to isolate use cases of the algorithm based on the number of days on CRRT (without another model). Therefore, testing splits were made of patients who were on CRRT for up to seven days, as well as more than seven days, with results reported for each sub-population.

### 2.2 Evaluation of model performance

**Fig. 2:**
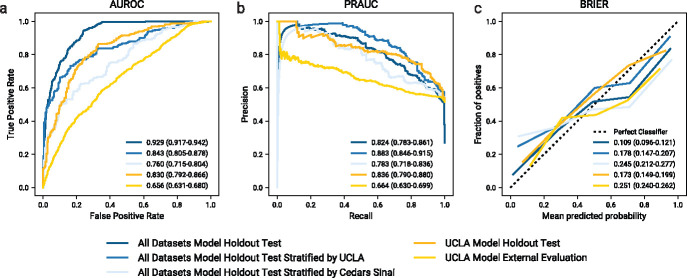
Model performance when predicting CRRT patient outcomes. Blue curves illustrate the performance of a model on the holdout test dataset (n=1,535) after training on a combination of UCLA: CRRT, Cedars: CRRT, and UCLA: Control cohorts (n=4,576); the darkest blue curve illustrates the overall performance on the holdout test set, while the lighter and lightest blue curves illustrate the stratified results on the UCLA: CRRT (n=430) and Cedars: CRRT (n=354) constituents of the test dataset. Yellow curves illustrate the performance of a model trained on single-institution data from UCLA: CRRT (n=1,270), evaluated on both an internal holdout test dataset (n=430) shown in darker yellow, and an external dataset from Cedars: CRRT (n=1,790) shown in lighter yellow. Reported statistics include point estimates as well as 95% confidence intervals obtained from 1,000 bootstrap iterations of the test dataset. a) Receiver operating characteristic curves for the prediction of CRRT outcome. Summarizing metric is the receiver operating characteristic area under the curve (ROCAUC). b) Precision curves for the prediction of CRRT outcome. Summarizing metric is the precision recall area under the curve (PRAUC). c) Calibration curves for the prediction of CRRT outcome. The summarizing metric is the Brier score.

We investigate multiple experiments that consider different combinations of cohort data (Methods 4.3), with optimal models after tuning documented in Extended Data A2. We define the most comprehensive model as the model we trained and evaluated on a combination of the UCLA: CRRT, Cedars: CRRT, and UCLA: Control cohorts, using only features shared across all cohorts (635 features); the stratified train, validation, and test splits consisted of 4,576, 1,517, and 1,535 samples, respectively. The inclusion of the control data imbalanced the proportion of outcomes in each split, with 27.0% recommended being put on CRRT. However, the isolated counts of UCLA: CRRT and Cedars: CRRT patients within the test split were more balanced at 430 (54.0% positive) and 354 (51.7% positive) patients, respectively. Classification performance on both the entire test split and cohort subgroups is illustrated in Figure 2 (see blue curves). The model achieves a receiver operating characteristic area under the curve (ROCAUC) of 0.929 (CI=0.917–0.942) and precision recall area under the curve (PRAUC) of 0.824 (CI=0.783–0.861), improving upon the uncertain CRRT outcomes observed in current clinical practice. Results of the isolated subgroups indicate better performance on the UCLA: CRRT cohort (ROCAUC: 0.843, CI=0.805–0.878; PRAUC: 0.883, CI=0.846–0.915) than the Cedars: CRRT cohort (ROCAUC: 0.760, CI=0.715–0.804; PRAUC: 0.783, CI=0.718–0.836). The calibration curves demonstrate a similar trend, with slight overestimation at high probabilities and underestimation at low probabilities.

### 2.3 Model and feature interpretation

**Fig. 3:**
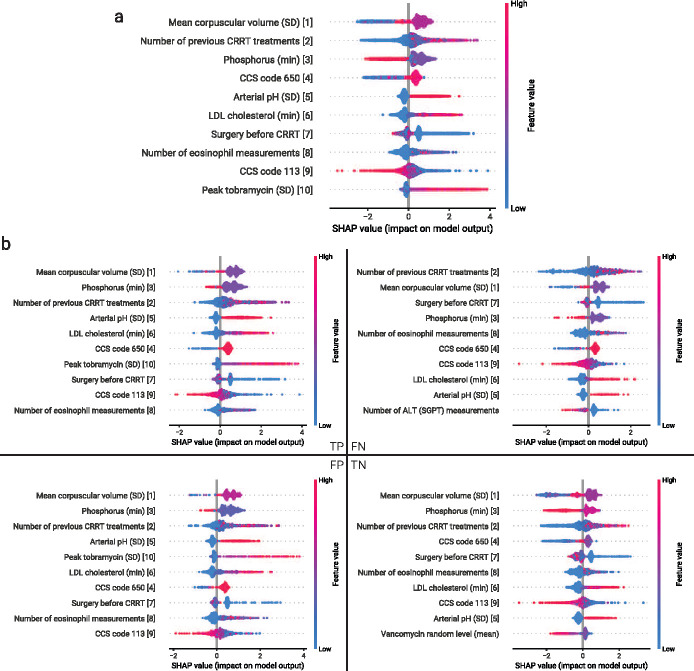
Model explanations for the model trained and evaluated on a combination of the UCLA: CRRT, Cedars: CRRT, and UCLA: Control cohorts, using only features that existed across all three cohorts (defined in Methods 2.2). SHAP values were evaluated using the holdout test set, in addition to patients that were on CRRT for more than 7 days (*N* = 4159 (46.5% recommending CRRT)). a) Ordered ranking of the ten most important features by average magnitude of SHAP values and direction of influence on output predictions. b) Ordered ranking of the ten most important features by average magnitude of SHAP values when isolating subpopulations of patients in the test set that were classified as true positives, true negatives, false positives, and false negatives. The corresponding rank of each feature in the ordered ranking of feature importance using the entire dataset is reported for each feature (for the top ten features).

We evaluated the Shapley additive explanations (SHAP) values of the optimal model defined in Section 2.2 on the entire test split (Figure 3a), as well as subsets based on a confusion matrix (Figure 3b). The top ten features for all patients have significant overlap with the top ten features for the subset of patients that were true positives (TPs), true negatives (TNs), false positives (FPs), and false negatives (FNs) (Fig. 3b). Notably, MCV stability maintained its role as the most important feature with the same patterns across TP, FP, and TN; however, decisions made for patients that were FN were mostly driven by the number of previous CRRT treatments. The feature value distribution of the top feature suggests that a moderate amount of fluctuation in mean corpuscular volume (MCV) over the 10-day window lends to a higher likelihood of recommending CRRT initiation, a highly variable MCV is unclear, and a low variability MCV lends to a lower likelihood of recommending CRRT. The second most important feature suggests that having previous CRRT treatments lends to a higher likelihood of recommending CRRT, as it may indicate previous survival and benefit from CRRT.

**Fig. 4:**
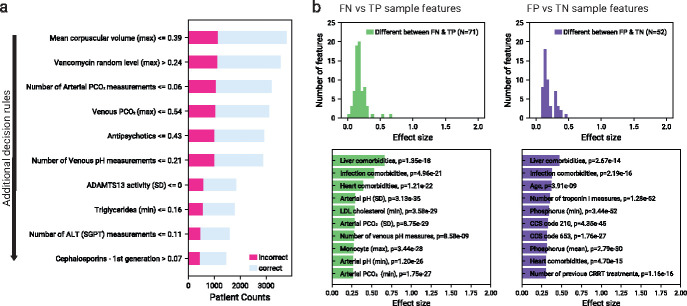
Error analysis for the model trained and evaluated on a combination of the UCLA: CRRT, Cedars: CRRT, and UCLA: Control cohorts, using only features that existed across all three cohorts (defined in Section 2.2). Analysis was performed on the holdout test set, in addition to patients that were on CRRT for more than 7 days (*N* = 4159 (46.5% recommending CRRT)). a) Top ten features that contributed to the majority of the errors. The threshold applied at each row operates on the resulting population after applying the respective threshold in the immediately above row. b) Summary of analysis of model randomness against feature noise. Effect sizes are shown for the features that are significantly different between false negative and true positive populations (left). Effect sizes are also shown for the features that are significantly different between false positive and true negative populations (right). Features with the top ten effect sizes are shown.

We also identified features that contributed to the majority of errors via model error analysis (Mealy) [17] and illustrated the path of the decision tree that contributed the most errors (Figure 4a). The maximum MCV contributed to the most errors, compared to the standard deviation of MCV, which was the most important feature from our SHAP analysis. No other top important feature for prediction was related to a top feature contributing to errors. Patients who had a higher concentration of vancomycin, an antibiotic, led to the second most amount of errors.

Lastly, we investigated if the feature distributions from Type I and II errors were statistically different from the subset of correctly predicted patients that shared the same true label (Figure 4b). The number of significantly different features between FN and FP patients from their correct counterparts is *N* = 71 and *N* = 52 respectively. A small subset of features appeared different for both cases, namely the presence of certain heart conditions, with indicators of liver comorbidities having the largest effect size. Some of the features with the largest effect size were also important factors driving predictive decisions, and few existed on the decision path of the largest number of errors in Fig. 4a. While it is not certain if the model uses the feature values that distinguished errors from their correct counterparts to discriminate between true and false predictions, the features are potential confounders that could use additional analyses.

### 2.4 Clinical considerations of an applied model

**Fig. 5:**
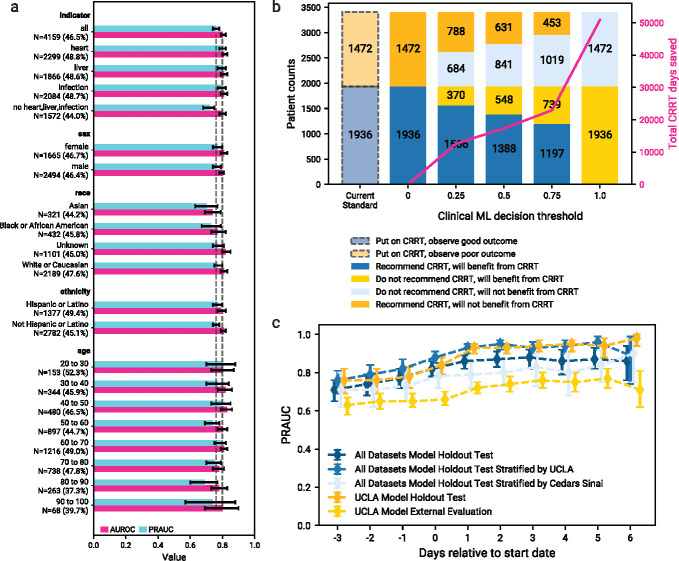
Additional analyses and evaluation. a) Performance after training on a combination of UCLA: CRRT, Cedars: CRRT, and UCLA: Control cohorts (n=4,576), measured by ROCAUC and PRAUC on a holdout test set (n=4,159) including patients who were on CRRT for more than seven days (model defined in Section 2.2). Results are also reported when applying the model to subgroups of the test set, categorized by disease indicator, sex, race, ethnicity, and age. b) Potential clinical impact of different operating thresholds (0.0, 0.25, 0.5, and 0.75, 1.0) compared to current clinical standards. Estimated savings when not putting patients on CRRT are shown at each threshold. c) Evaluation of the same models and datasets described in 2, when using features from shifted windows relative to the start date. The observation window is shifted from three days before starting CRRT to six days after starting CRRT, and the models are evaluated (without re-training) on the subset of test patients who have available features for each shifted window.

Figure 5a visualizes the performance of the optimal model defined in Section 2.2 on different subgroups of patients (Methods 4.4) from the the holdout test set, in addition to patients that were on CRRT for more than 7 days (*N* = 4159, 46.5% recommending CRRT). False negative rates, false positive rates, and confusion matrices for each subgroup are illustrated in Extended Data A1. Predictive performance is higher for the patients with heart, liver, or infection indications, over those without these indications, which had slightly more imbalanced outcomes. We did not observe a difference in performance between sex or ethnicity groups, despite the larger imbalance of outcomes in Non-Hispanic or Latino patients. The model performs least accurately for Asian patients, the least balanced in outcome (44.2%), and most accurately for White or Caucasian patients, the most balanced (47.6%). We observe a degradation of performance for older patients, which may be tied to the trend in the increasing degree of outcome imbalance in those groups. While typically less balanced datasets lead to worse performance, the trend between the balance of outcome and the performance needs to be explored, particularly causes of imbalance and explanations of potentially poor performance.

When the model is used as a support tool in a decision-making process, the prediction threshold should be adjusted to take into consideration operational factors. We demonstrate outcomes under different operating thresholds and the projected estimate of number of days saved from not placing patients on CRRT in Figure 5b. The current standard of clinical practice for patients considered for CRRT would be to always place patients on CRRT, which is the equivalent of a threshold of 0. As the threshold for placing patients on CRRT increases, fewer patients are placed on CRRT. Stricter thresholds lead to not recommending treatment initiation despite potential benefit, but also not recommending treatment to those who would not benefit. Even with a moderately strict threshold of 0.25, we project that a hospital system might save on the order of over ten thousand treatment days. Our analyses provide insight into how our model might change over different thresholds; however, we will need to develop a procedure for determining the optimal operating point for any institution that would use our model in assisting with decision-making.

We also evaluated our model on a rolling basis (without retraining), on patients treated with CRRT for a maximum of seven days (Figure 1c). This analysis allows for the evaluation on data after patients started CRRT (which would change their biological processes), without leaking information into model training. We observed that the model improved as the data became more topical to the patient’s outcome, suggesting that it learned meaningful relationships in the data prior to starting CRRT that maintained importance (Figure 5c). This observation suggests that a dynamic model that evaluates on a day-by-day basis may be more clinically useful and a natural next step. Consequently, we believe multiple models may be most helpful when used together to assist with this clinical decision-making task: deciding if a patient should initiate CRRT, predicting how many days of CRRT a patient would require to benefit, and finally if that patient should remain on CRRT on a day-by-day basis after initiating treatment.

### 2.5 Comparison between single and multi-site models

We also provide examples of externally validated local solutions via the cross institutional application of a model trained on a single institution. Models are trained, validated, and tested on the patients and features from a single cohort; the tuned models are then externally tested (with imputation of missing features) on all patients from another cohort. Figure 2 illustrates the performance of a model trained and validated on data from the UCLA: CRRT cohort (n=1,270 and n=416 respectively; both 52.6% positive), evaluated both on the internal test set (n=430; 52.6% positive) and external Cedars: CRRT test set (n=1,790; 51.6% positive). When applying the optimal model (Extended Data A2) to the internal test dataset, the performance is comparable to that of the comprehensive model in Section 2.2 (ROCAUC: 0.830, CI=0.792–0.866; PRAUC: 0.836, CI=0.790–0.880); however, performance when externally testing on the Cedars: CRRT cohort is worse than the comprehensive model (ROCAUC: 0.656, CI=0.631–0.680; PRAUC: 0.664, CI=0.630–0.699). A similar trend is observed in Figure 5c, whereby the model trained on data from a single institution is comparable to the performance of the comprehensive model when evaluating data from the same institution but with lower performance on the external data.

## 3 Discussion

We present for the first time, a machine learning model that predicts whether a patient should start CRRT. The current lack of consensus on clinical guidelines around CRRT initiation and pressure from family and consultants contribute to the often inappropriate use of CRRT. Although APACHE II [18] and SOFA [19] provide baselines for ICU mortality and have been proposed for CRRT use, there is a lack of specific models for solving issues regarding decision-making for CRRT initiation, and outcomes clearly remain suboptimal [9, 14, 15]. Ultimately, while many factors must be considered to determine the appropriateness of starting CRRT, we show that using EHR information in our model can help discern patients who will clinically benefit from this intervention, particularly within a 7-day timeframe. Our overall performance, as measured by ROCAUC and PRAUC, is a significant improvement over the current reported rates of only 37–55% of individuals surviving through this intervention [9–11].

Our model interpretation also provides an initial understanding of what features drive predictions and the nature of errors. Interestingly, we found statistical descriptors of MCV were responsible in both cases, which may reflect the stability of the patient before CRRT initiation or if they received a blood transfusion [20]. Future exploration of the role of key variables like MCV among other emergent features from our analyses and their causal connection to CRRT outcomes could contribute to objective clinical guidelines for CRRT initiation. Removing important features that are not strongly considered in current clinical practice may be a future direction.

Our model trained on multi-institutional data demonstrates the promising value of applying data-driven methods to CRRT, yet the labels and collected data may be biased by the individual medical provider and institution’s protocol. The generalizability of the model still remains in question and could be evaluated with larger and more diverse datasets. On the other hand, even though generalizable models trained on data across multiple institutions are desirable for knowledge discovery, we also show that local solutions may be adequate or even better than general solutions if they are deployed in appropriate contexts, especially if resources are constrained (e.g., limited access to training and evaluation data). Moreover, our analysis demonstrated the importance of incorporating control groups in the model development process, as they enabled the model to discern patterns distinguishing between mortality (while not on CRRT) and cases where patients do not benefit from CRRT.

We also highlight additional important considerations for using such a model in a more specific manner than a general calculator such as SOFA. Subgroup analysis provides insight into bias and the need for further assessment across important groups such as race and causes of inconsistent performance. Additionally, as we only use historical data of a patient before they begin CRRT treatment, our model is unaware of possible changes that might occur after their treatment start date that might affect their outcome. However, the rolling window analysis demonstrates that the model performs better as the data used for inference gets closer to each patient’s endpoint. We can intuitively understand that the model makes the best prediction it can at “day zero” with the available information, even though with updated data the model may predict differently (and more accurately). The findings suggest that our data processing and predictive pipelines are appropriate for this task, but also that further improvement may be discovered with a dynamic model, or separate models for predicting CRRT initiation and CRRT termination.

Lastly, we demonstrate the value of our model in terms of resource-savings measured by preventable days on CRRT, which are significant even at a low threshold; the choice of threshold also presents another aspect of tuning and granularity that our model provides over regular calculators. Due to natural variability in the decision making process, we believe that tailoring to particular institutions and healthcare providers based on their needs could be beneficial. It is also important to analyze such sources of variability to provide insight into a more precise model; for example, comfort care patients who were only put on CRRT to keep the patient alive long enough for a family member to visit would still be considered a negative outcome in the current model (our model and current dataset do not consider such alternative definitions of a positive outcome). A prospective detailed case analysis to compare decision-making side-by-side with our predictive model could further shed light into decision-making around CRRT initiation and improve this data-driven approach.

Overall, this paper raises considerations for the future development of machine learning for CRRT, and the potential for further data collection to support additional evaluations. We present the utility of machine learning for CRRT and identify a space of features to consider; the next step is to work towards clinical translation. Simpler versions of this predictive model, by honing in on important features for different subgroups or site-specific features, could make the model more easily understood by clinicians, as well as accessible to a wider range of institutions.

## 4 Methods

### 4.1 Data

Our dataset consists of three cohorts across two different hospital systems: UCLA and Cedars-Sinai Medical Center. The first cohort is the UCLA: CRRT population (*N* = 4,161 patients), which contains adult (21 years of age or older at the start date of treatment) patients treated at UCLA that were all placed on CRRT between 2014 and 2021. The second is the UCLA: Control population (*N* = 4,725 patients), which contains adult patients treated at UCLA between 2005 and 2022 who were not placed on CRRT but matched to individuals in the UCLA: CRRT cohort based on race, ethnicity, age, sex, and disease status (via the Charlson Comorbidity Index) via cosine similarity. The last cohort is the Cedars: CRRT population (*N* = 3,263 patients), which contains adult patients treated at Cedars who received CRRT between 2016 and 2021.

Our outcome of interest is whether a patient should be placed on CRRT. For the CRRT cohorts at UCLA and Cedars, we construct the final binary target from four clinical outcomes: Recover Renal Function, Transition to Hemodialysis, Comfort Care, and Expired. The former two represent conditions in which we would recommend CRRT; the patient’s kidneys either completely recovered or the patient was able to stabilize on CRRT and could continue with hemodialysis. The latter two represent conditions in which we would *not* recommend CRRT; the patient did not stabilize on CRRT and continued to end of life care or passed away while on treatment. All patients in the UCLA control cohort should not be placed on CRRT and are labeled as such.

Features describing demographics, vitals, medications, labs, medical problems, and procedures are available for all three cohorts. Demographics were collected once and consisted of information regarding age, sex, race, and ethnicity. Basic descriptive statistics (demographics, outcome variables) for all three cohorts are illustrated in Table A1. The remaining longitudinal features were collected at multiple time points, before and after starting CRRT. The diagnoses and medical problems are documented using ICD-10 codes, along with the respective dates of diagnosis and dates of entry. The procedures are documented using CPT codes and the dates of procedures. Vitals (e.g., temperature, weight, height, systolic/diastolic blood pressure, respiration rate, pulse oximetry, heart rate) are described via numeric value and observation time. Medications are recorded using pharmaceutical subclass identifiers, along with the order date. Lastly, labs are described with the name of the target component or specimen, the value of the result, and the order date. While being a data-driven approach, clinicians were consulted regarding the feature list and approved the features used in the analysis.

### 4.2 Data Preprocessing

The electronic health record (EHR) data are processed for downstream use in machine-learning pipelines. We define each sample as a (patient, treatment) pair with unique outcomes, as a given individual may have more than one treatment. For samples in the UCLA: Control cohort, we construct outcomes and randomly choose a procedure date to act as a proxy for a treatment start date. For each sample, we load and aggregate all longitudinal data over a predefined window of *d* days. Furthermore, we filter the training and validation samples to those who were on CRRT for a maximum of seven days. The continuous features over the window are aggregated to the minimum, maximum, mean, standard deviation, skew, and number of measurements. The categorical features over the window are aggregated to the count of occurrences of each category. All ICD codes are converted to Clinical Classifications Software (CCS) codes in order to reduce the number of categories [21], and all procedures are converted to Current Procedural Terminology (CPT) codes [22, 23]. We align the names of all vital, medication, and lab names across all cohorts in reference to the data contained in the UCLA: CRRT cohort. For vitals and labs, we also unify the units. Additionally, if any values are recorded as ranges bounded on one side, we assume the value to be the bound (e.g., a value of > 4 is assumed to be 4). We then combine the outcomes, the aggregated longitudinal features, and the static features (patient demographic information). We engineer a categorical feature from CCS codes that indicates common reasons for a patient requiring CRRT such as whether a patient has an indication of liver problems (CCS codes: 6, 16, 149, 150, 151, 222), heart problems (CCS codes: 96, 97, 100, 101, 102, 103, 104, 105, 106, 107, 108, 109, 114, 115, 116, 117), severe infection (CCS codes: 1, 2, 3, 4, 5, 7, 8, 249), or none of the previous three.

### 4.3 Model Training and Evaluation

#### 4.3.1 Experiments

We define an experiment as the full procedure required to train and evaluate a unique predictive model. For each experiment, we must specify a cohort or combination of cohorts on which we would like to train and validate the model. The cohort(s) chosen are divided into train and validation splits using 60/20% of the data, respectively, with the remaining 20% as an internal test split. The entirety of any remaining cohort is used for external testing. When testing on the UCLA: Control cohort, the data is combined with the test data from the UCLA: CRRT cohort to allow the evaluation of performance metrics (all samples in the control cohort have the same label). When training on multiple CRRT cohorts, performance is also evaluated on the isolated samples from each constituent CRRT cohort.

We explore all the following patterns to train and evaluate our methodology: 1) Experiment 1: train on UCLA: CRRT, evaluate on UCLA: CRRT, Cedars: CRRT, UCLA: Control; 2) Experiment 2: train on Cedars: CRRT, evaluate on Cedars: CRRT, UCLA: CRRT, UCLA: Control; 3) Experiment 3: train on UCLA: CRRT combined with Cedars: CRRT, evaluate on CRRT combined with Cedars: CRRT, UCLA: Control; and 4) Experiment 4: train on UCLA: CRRT combined with Cedars: CRRT combined with UCLA: Control, evaluate on CRRT combined with Cedars: CRRT combined with UCLA: Control. Experiments 1–4 are first conducted using the original number of features available in the training split. When training on multiple cohorts (Experiments 3 and 4), the total number of features comprises the union of the features of the individual cohorts. We also re-run Experiments 1–4 with an extra feature selection step that reduces the feature set to those that exist in all three cohorts. Lastly, we re-run Experiments 1–3 after reducing the feature set to those that exist in the UCLA: CRRT and Cedars: CRRT cohorts. While the main body of the paper focuses on Experiment 1 (using the original training features) and Experiment 4 (using the features that exist in all three cohorts), results for Experiment 2 (using the original training features) and Experiment 3 (using the features that exist in the UCLA: CRRT and Cedars: CRRT cohorts) are illustrated in Extended Data A2, A3, A4, A5, A6, A7, A8.

#### 4.3.2 Model Hyperparameters and Tuning

Each experiment yields the optimal model of a grid of hyperparameters. Candidate models *m* include: light gradient-boosting machine (LGBM), extreme gradient-boosted decision tree (XGB), random forest (RF), or logistic regression (LR). Each model type has its own (possibly different) hyperparameters that are also tuned as a part of the same grid. We select the look-back window size *w*, or the number of days before each patient’s treatment start date from which to aggregate data, from the options of 1, 2, 3, 4, 5, 6, 7, 10, and 14 days. We select an imputation method between *simple* imputation (which we use to refer to mean imputation on continuous or quantitative features and mode imputation on categorical or qualitative features) and *k* nearest neighbors. Note that the imputation method is trained on the training split; therefore, if features in the training cohort do not exist in the testing cohort, then entire features may be imputed in the testing cohort. After imputation, the data are scaled between 0 and 1 using minimum-maximum scaling. We also decide on a feature selection procedure based on the Pearson correlation coefficient between each feature and the target variable by selecting the top features (high correlation to the outcome) for a particular number of features (*k*-best, where *k* ∈ {3,8,13,18}) or by using a correlation threshold (*ρ*, where *ρ* ∈ [0.01,0.09] at intervals of 0.005). For a detailed breakdown of these hyperparameters, refer to Supplementary Information 1. We run *t*=200 trials of tuning by randomly sampling from the above hyperparameter grid *t* times. For each trial *t*_*j*_, we load the data from the designated cohorts and divide it into the training, validation, and testing portions. We aggregate these three splits over the look-back window of *w*_*j*_ days. The imputation and feature selection procedures are trained only on the training split but applied to all three splits. We train the selected model *m*_*j*_ on the train split and then evaluate its performance on the validation split. We then select the model with the highest receiver operating characteristic area under the curve (ROCAUC) on the validation dataset and evaluate its performance on the testing dataset. Performance is measured by ROCAUC, PRAUC, Brier score, precision, recall, specificity, and F1 score. Confidence intervals are obtained through 1,000 bootstrap iterations of the test split.

The optimal hyperparameters for all experiments after tuning are shown in Table A2. Simple imputation and feature selection using a correlation threshold are optimal for all experiments. Table A2 also describes the number of raw (before processing) and engineered (and after processing) features available when using the optimal hyperparameters for each experiment, before and after training. The features available for Experiments 1–4 are 2,350, 1,050, 3,044, and 3,238 respectively. When reducing the feature set to the intersection of the features between all three cohorts, the features available for Experiments 1–4 are 575, 595, 635, and 635, respectively. Lastly, when reducing the features to the intersection of the features between the UCLA: CRRT and Cedars: CRRT cohorts, the remaining features available for Experiments 1–3 are 651, 703, and 696 respectively.

### 4.4 Subpopulation Analysis

In addition to evaluating on the whole test dataset, we evaluate our model on subgroups of the dataset based on different characteristics. Evaluations in this analysis are performed on the combined set of patients who were on CRRT for up to seven days and more than seven days to reflect the performance of the model in a realistic setting. One set of characteristics includes common medical reasons for requiring CRRT (identified via ICD code diagnoses), including heart issues, liver issues, and infection. These are some of the most common conditions in which a patient might be hemodynamically unstable and require CRRT. For a list of codes we use for each indicator, refer to Section 4.2. Note that these groups are not mutually exclusive; for instance, a patient may have a heart condition and also be suffering from a severe infection. Other characteristics are based on sex, race, ethnicity, and age groups.

### 4.5 Rolling Window Analysis

Each CRRT patient (as this scenario does not apply to controls who never started CRRT) receives treatment for a different number of days, so we may be making predictions on variable outcome horizons from the start of treatment. During that time, patients are under active care, and their condition may fluctuate due to their own physiologic processes or because of direct medical intervention. A patient’s outcome may also stem from events that occurred *after* they began treatment, meaning we would not be able to capture that data for new patients who have not been on CRRT. Thus, despite the dynamic nature of CRRT, if the goal of our model is to predict if a patient should start CRRT, then we cannot train our model on post-start data, otherwise we would be leaking information. However, we can *evaluate* our model on future data. We, therefore, implement a *rolling window analysis* (visualized in Fig. 1a), evaluating the model (without retraining) on a set of *w* day windows that each starts a day later from the previous window. By monitoring metrics over each window, we can analyze how the predictions and the performance of the model change as the data nears the outcome horizon (i.e., end of CRRT). If we observe an improvement in performance as the model is evaluated on data closer to the outcome horizon without retraining, we can infer that: 1) outcomes influenced by events after they start CRRT; and 2) the model has learned meaningful information, and evaluating on the changing information has led to updated predictions for some patients on later windows. Similar conclusions can be drawn if we observe a decrease in performance as the model is evaluated on data further from the outcome horizon without retraining. We perform the rolling window analysis on patients on CRRT for a maximum of seven days because for patients with far-away outcome horizons, data after but near the start date may still be uninformative.

Note that the rolling window analysis looks at contiguous days of data at once and evaluates a model trained on data before the start of treatment. This procedure is hence completely unaware of previous days or how measurements change over time which would be used in an ongoing day-by-day predictive task.

### 4.6 Explainability

It is critical when building machine learning models for clinical tasks that the model is understandable to promote transparency, correctness, and trust. We must understand *why* a model makes its decision, not just how and where it makes the most errors. To this end, in addition to technical performance metrics, we plot the feature importance of the model applied to the entire test split (as well as for the subset of true positive, false positive, true negative, and false negative instances) via SHAP [24]. We also visualize the features that contribute the most to error via Mealy, which trains a secondary decision tree classifier to predict if the original model will output an incorrect or correct prediction [17].

To establish a deeper understanding of our model, we further evaluate if the model is learning distinctions between patients with incorrectly and correctly predicted outcomes. For each feature, we compare pairs of incorrectly and correctly predicted populations in the confusion matrix (i.e., false negative vs. true positive, false positive vs. true negative) and test if the distributions of that feature are statistically significantly different between the two groups. We employ a different statistical test and effect size formula depending on the type of feature. For a detailed breakdown of statistical tests and effect size formulas used, refer to Table 1. If we reject, we reason that the model may be using the feature to incorrectly distinguish the populations due to factors such as confounding.

### 4.7 Computational Software

We implemented our procedure in Python 3.9.16. We primarily use the NumPy 1.23.0 [25] and pandas 1.5.3 [26] packages for loading and manipulating our datasets. Models are implemented via lightgbm 3.3.5 [27] for an implementation of LGBM, xgboost 1.7.4 [28] for an implementation of XGB, and Scikit-learn 1.2.2 [29] for all other models. We use the Optuna 3.2.0 [30] framework for model tuning and the algorithm for sampling from the hyperparameter grid. Statistical tests are computed using the SciPy 1.10.1 and Scikit-learn 1.2.2 packages. We use Shapley additive explanations (SHAP) 0.42.0 for generating feature importance and explaining model decision-making on particular samples. Lastly, we use the Mealy 0.2.5 package for visualizing model errors.

### 4.8 Statistical Analysis

All statistical analyses are performed using Python 3.9.16 and SciPy 1.10.1. When comparing between-group differences in continuous variables, the Shapiro-Wilk test is first used to test for normality; then, normally distributed data are compared using the Student’s t-test, and non-normally distributed data are compared using the Wilcoxon rank-sum test. Hedges’ G statistic is used to describe the effect size. Binary-categorical variables are compared using Fisher’s exact test, and Cohen’s h is used to describe effect size. Multi-categorical variables are compared using the Chi-squared test, and Cramer’s V is used to describe effect size. All statistical tests are evaluated at a significance level of p=0.05, with the Bonferroni correction for multiple testing.

## Figures and Tables

**Table 1:** Breakdown of Statistical Tests Used Based on Feature Characteristics

	Variable Type	Statistical Test Name	Effect Size Formula

Continuous	$X\sim {𝒩}^{1}$ ^1^	*t* test	Hedges *g*
	$x≁{𝒩}^{1}$ ^1^	Mann-Whitney *U* test	
Categorical	Binary	Fisher’s Exact test	Cohen’s *h*
	Multicategory	$\chi^{2}$ test	Cramer’s *v*

1 𝒩 indicates the Normal or Gaussian distribution.

**Algorithm 1:** Train, tune, and evaluate a model to predict if a patient should be placed on CRRT.

1:	$D_{\text{train+val}\;}⇐$ choose from {UCLA: CRRT, UCLA: Control, Cedars: CRRT }	
2:	$D_{\text{test}\;}⇐$ choose from {UCLA: CRRT, UCLA: Control, Cedars: CRRT }	
3:	**if** $D_{\text{train+val}\;}=D_{\text{test}\;}$ **then**	
4:	$D_{\text{train}},D_{\text{val}},D_{\text{test}}⇐D_{\text{train+val}}\text{*}\left[0.6,0.2,0.2\right]$	▷ Split dataset into 60/20/20% respectively.
5:	**else**	
6:	$D_{\text{train}},D_{\text{val}}⇐D_{\text{train}\;\text{+val}}\text{*}\left[0.8,0.2\right]$	▷ Split dataset into 80/20% respectively.
7:	**end if**	
8:	w⇐{1,2,3,4,5,6,7,10,14} days	▷ look-back window size
9:	m⇐{lgbm,xgb,rf}	▷ model type
10:	i⇐{mean/mode,knearestneighbors}	▷ imputation method
11:	f⇐{kbest,correlationthreshold}	▷ feature selection method
12:	ℍ⇐{w,m,i,f}	▷ Set hyperparameter grid
13:	**function** Tuning $\left(ℍ,D_{\text{train}},D_{\text{val}}\right)$	
14:	metrics ⇐ {}	
15:	**for** $w_{j},m_{j},i_{j},f_{j}\in ℍ$ **do**	
16:	Train $m_{j}\left(f_{j}\left(i_{j}\left(D_{\text{train}}\right)\right)\right)$	
17:	$\text{metrics}\;\overset{+}{⇐}\;\text{Evaluate}\;m_{j}\left(f_{j}\left(i_{j}\left(D_{\text{val}\;}\right)\right)\right)$	
18:	**end for**	
19:	$m^{\text{*}}⇐m_{j}$ such that corresponding metric is max/min (best)	
20:	**return** m*	
21:	**end function**	
22:	$m^{\text{*}}⇐\text{Tuning}\left(ℍ,D_{\text{train}\;},D_{\text{val}\;}\right)$	
23:	metrics ⇐ Evaluate $m^{\text{*}}\left(D_{\text{test}}\right)$	
24:	subpopulations ⇐ (heart, liver, infection) × (male, female, race)	
25:	**for** subpopulation in subpopulations **do**	
26:	metrics by subpopulation ⇐ Evaluate $m^{\text{*}}(D\text{test}\;{\;}^{\text{*}}\;\text{subpopulation})$	
27:	**end for**	

**Fig. A1:**
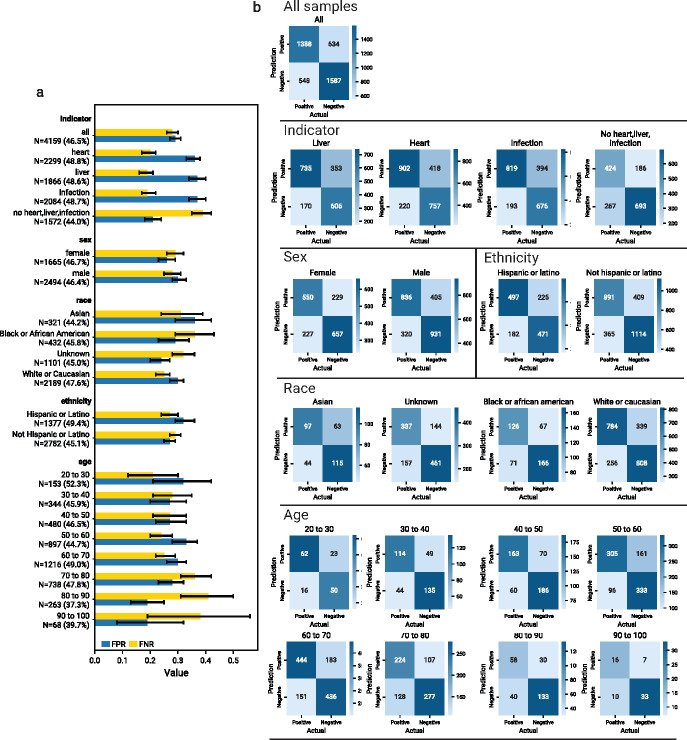
Bias analysis for the model trained and evaluated on a combination of the UCLA: CRRT, Cedars: CRRT, and UCLA: Control cohorts, using only features that existed across all three cohorts (defined in Section 2.2). a) False positive rate and false negative rates are reported for a holdout test set (n=4,159) including patients who were on CRRT for more than seven days. False positive rates and false negative rates are also reported when applying the model to subgroups of the test set, categorized by disease indicator, sex, race, ethnicity, and age. b) Confusion matrices at a decision threshold of 0.5 when applying the model to same subgroups of the test set.

**Fig. A2:**
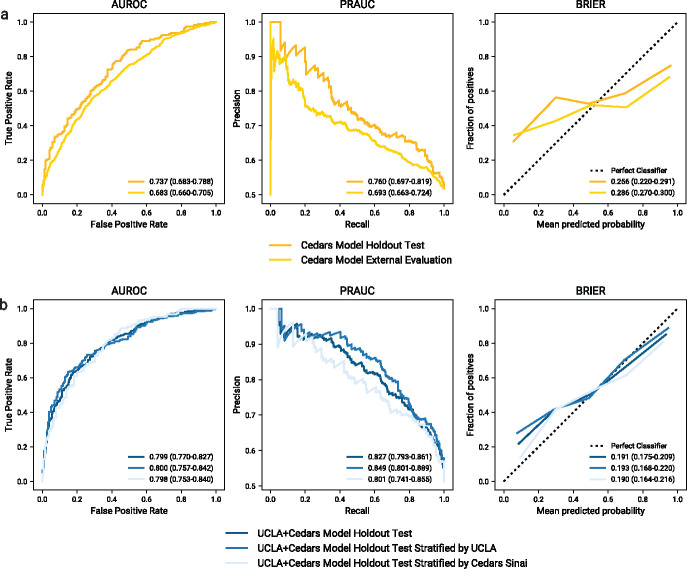
Model performance when predicting CRRT patient outcomes. The first column illustrates receiver operating characteristic curves for the prediction of CRRT outcome, with ROCAUC as the summarizing metrix. The second column illustrates precision curves for the prediction of CRRT outcome, with PRAUC as the summarizing metric. The third column illustrates calibration curves for the prediction of CRRT outcome, with the Brier score as the summarizing metrix. The reported statistics include point estimates as well as 95% confidence intervals obtained from 1,000 bootstrap iterations of the test dataset. a) The performance of a model trained on single-institution data from Cedars: CRRT (n=1,069), evaluated on both an internal holdout test dataset (n=363) shown in darker yellow, and an external dataset from UCLA: CRRT (n=2,122) shown in lighter yellow. b) Performance of a model on the holdout test dataset (n=803) after training on a combination of UCLA: CRRT and Cedars: CRRT cohorts (n=2,343); the darkest blue curve illustrates the overall performance on the holdout test set, while the lighter and lightest blue curves illustrate the stratified results on the UCLA: CRRT (n=431) and Cedars: CRRT (n=372) constituents of the test dataset.

**Fig. A3:**
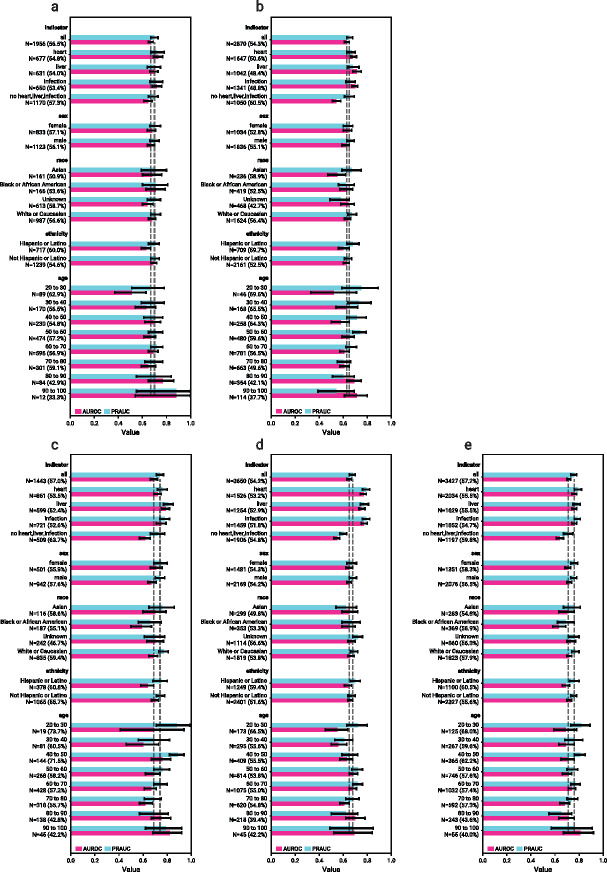
Performance measured by ROCAUC and PRAUC when applying models to subgroups of respective test sets, categorized by disease indicator, sex, race, ethnicity, and age. All test sets include patients who were on CRRT for more than seven days. a) Performance after training on data from UCLA: CRRT (n=1,270), and evaluating on a holdout test set (n=1,956). b) Performance after training on data from UCLA: CRRT (n=1,270), and evaluating on an external test set from Cedars: CRRT (n=2,870). c) Performance after training on data from Cedars: CRRT (n=1,069), and evaluating on a holdout test set (n=1,443). d) Performance after training on data from Cedars: CRRT (n=1,069), and evaluating on an external test set from UCLA: CRRT (n=3,650). e) Performance after training on a combination of data from UCLA: CRRT and Cedars: CRRT (n=2,343), and evaluating on a holdout test set (n=3,427).

**Fig. A4:**
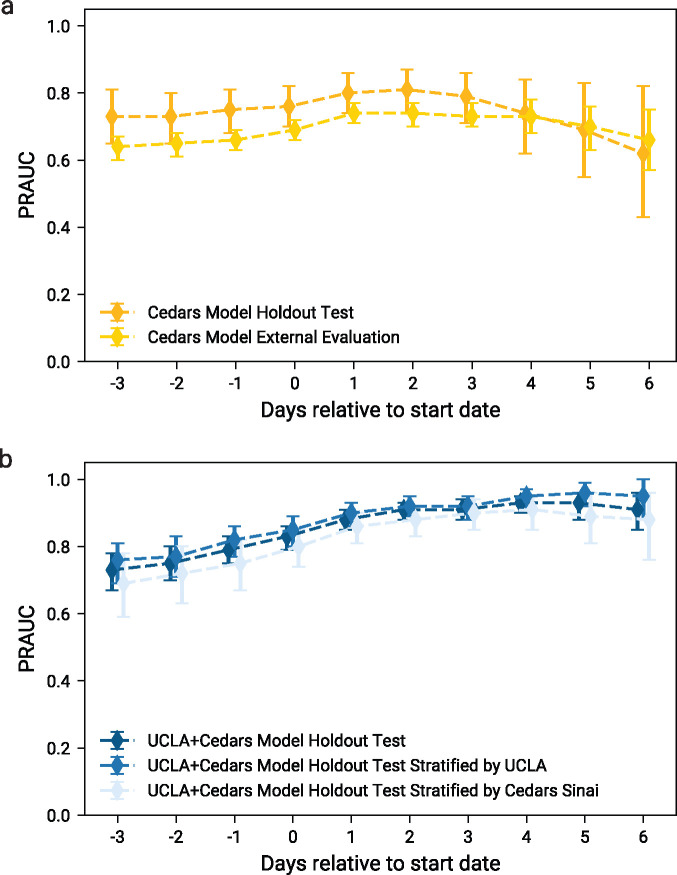
Model evaluation when using features from shifted windows between three days before and six days after the start date. All test sets are limited to patients who were on CRRT for within seven days. a) Model trained on data from Cedars: CRRT (n=1,069), evaluated on both an internal holdout test dataset (n=363) shown in darker yellow, and an external dataset from Cedars: CRRT (n=2,122) shown in lighter yellow. b) Model trained on a combination of data from UCLA: CRRT and Cedars: CRRT (n=2,343); the darkest blue curve illustrates the overall performance on the holdout test set (n=803), while the lighter and lightest blue curves illustrate the stratified results on the UCLA: CRRT (n=431) and Cedars: CRRT (n=372) constituents of the test dataset

**Fig. A5:**
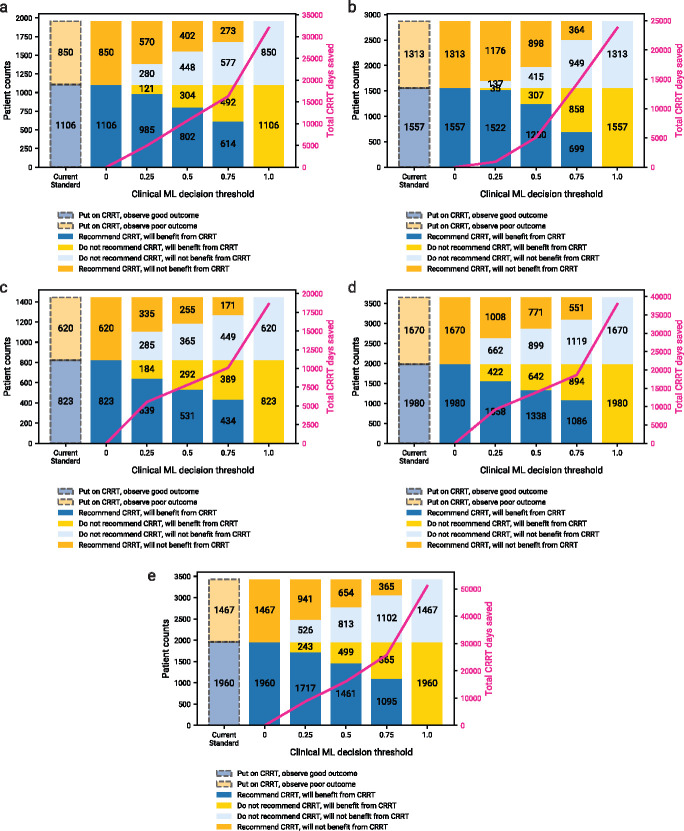
Potential clinical impact of different operating thresholds (0.0, 0.25, 0.5, and 0.75, 1.0) compared to current clinical standards. Estimated savings when not putting patients on CRRT are shown at each threshold. All test sets include patients who were on CRRT for more than seven days. a) Model trained on data from UCLA: CRRT (n=1,270), and evaluated on a holdout test set (n=1,956). b) Model trained on data from UCLA: CRRT (n=1,270), and evaluated on an external test set from Cedars: CRRT (n=2,870). c) Model trained on data from Cedars: CRRT (n=1,069), and evaluated on a holdout test set (n=1,443). d) Model trained on data from Cedars: CRRT (n=1,069), and evaluated on an external test set from UCLA: CRRT (n=3,650). e) Model trained on a combination of data from UCLA: CRRT and Cedars: CRRT (n=2,343), and evaluated on a holdout test set (n=3,427).

**Fig. A6:**
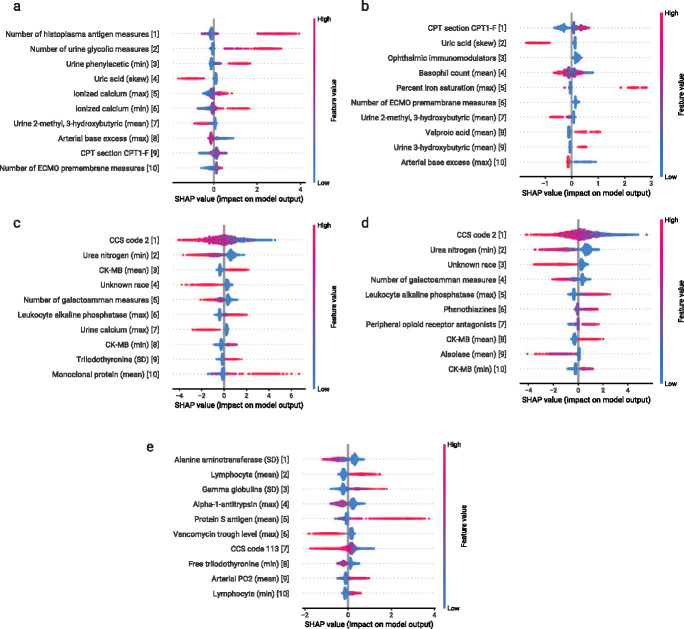
Ordered ranking of the ten most important features by average magnitude of SHAP values and direction of influence on output predictions. SHAP values were evaluated using holdout test sets that include patients who were on CRRT for more than seven days. a) Model trained on data from UCLA: CRRT (n=1,270), and evaluated on a holdout test set (n=1,956). b) Model trained on data from UCLA: CRRT (n=1,270), and evaluated on an external test set from Cedars: CRRT (n=2,870). c) Model trained on data from Cedars: CRRT (n=1,069), and evaluated on a holdout test set (n=1,443). d) Model trained on data from Cedars: CRRT (n=1,069), and evaluated on an external test set from UCLA: CRRT (n=3,650). e) Model trained on a combination of data from UCLA: CRRT and Cedars: CRRT (n=2,343), and evaluated on a holdout test set (n=3,427).

**Fig. A7:**
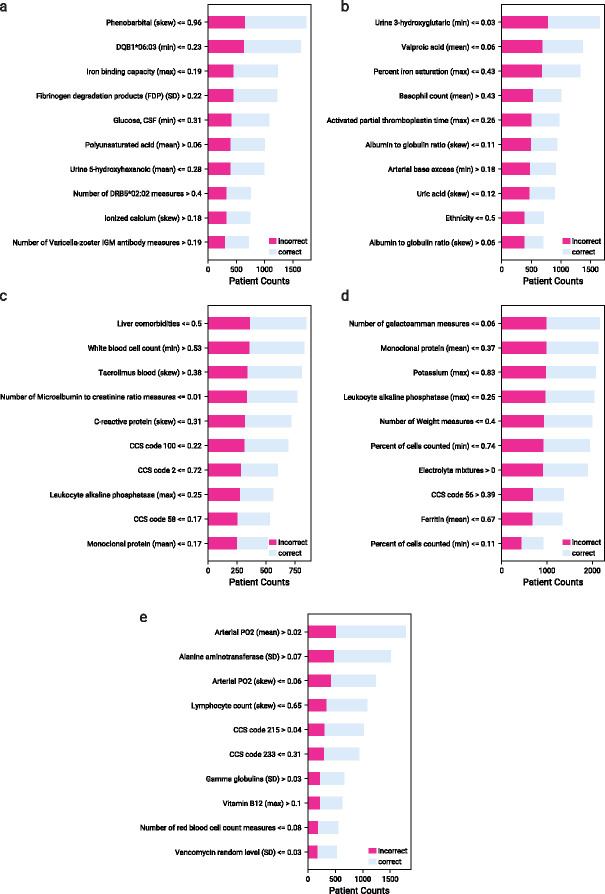
Top ten features that contributed to the majority of the errors. The threshold applied at each row operates on the resulting population after applying the respective threshold in the immediately above row. Error analysis was performed on holdout test sets that include patients who were on CRRT for more than seven days. a) Model trained on data from UCLA: CRRT (n=1,270), and evaluated on a holdout test set (n=1,956). b) Model trained on data from UCLA: CRRT (n=1,270), and evaluated on an external test set from Cedars: CRRT (n=2,870). c) Model trained on data from Cedars: CRRT (n=1,069), and evaluated on a holdout test set (n=1,443). d) Model trained on data from Cedars: CRRT (n=1,069), and evaluated on an external test set from UCLA: CRRT (n=3,650). e) Model trained on a combination of data from UCLA: CRRT and Cedars: CRRT (n=2,343), and evaluated on a holdout test set (n=3,427).

**Fig. A8:**
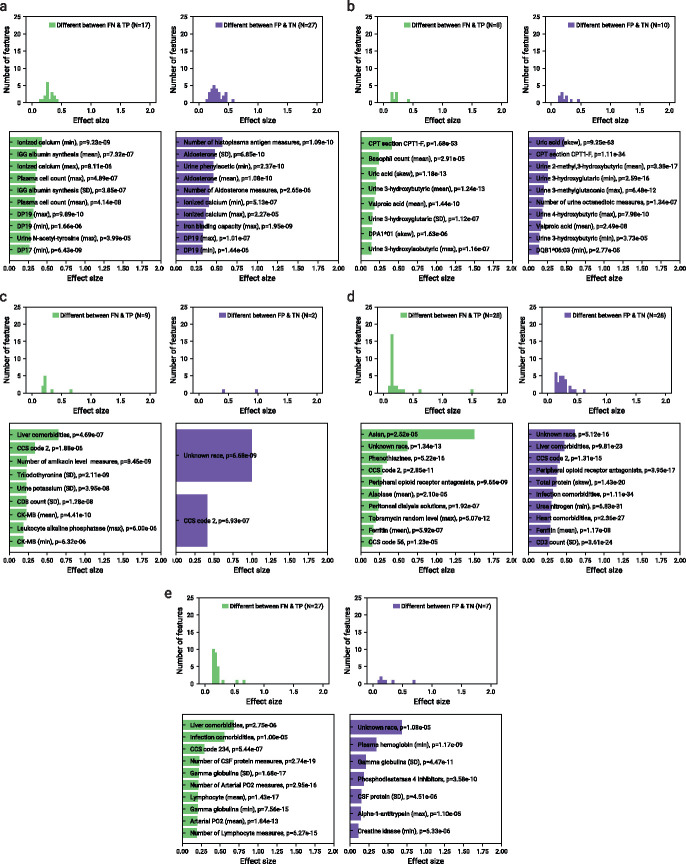
Summary of analysis of model randomness against feature noise. Effect sizes are shown for the features that are significantly different between false negative and true positive populations (green). Effect sizes are also shown for the features that are significantly different between false positive and true negative populations (purple). Features with the top ten effect sizes are shown. Analysis was performed on holdout test sets that include patients who were on CRRT for more than seven days. a) Model trained on data from UCLA: CRRT (n=1,270), and evaluated on a holdout test set (n=1,956). b) Model trained on data from UCLA: CRRT (n=1,270), and evaluated on an external test set from Cedars: CRRT (n=2,870). c) Model trained on data from Cedars: CRRT (n=1,069), and evaluated on a holdout test set (n=1,443). d) Model trained on data from Cedars: CRRT (n=1,069), and evaluated on an external test set from UCLA: CRRT (n=3,650). e) Model trained on a combination of data from UCLA: CRRT and Cedars: CRRT (n=2,343), and evaluated on a holdout test set (n=3,427).

## Data Availability

IRB approval was obtained to get de-identified data from UCLA and Cedars Sinai Medical Center. The de-identified EHR data will be uploaded to Dryad and will be compliant with Nature’s requirements and expectations.

## Appendix A Extended Data

**Table A1:** Description of cohort outcomes and demographics. Age data is missing from 36 patients in the UCLA: CRRT cohort. Age, sex, ethnicity, and race data is missing from 206 patients in the Cedars: CRRT cohort. Note that comorbidities are identified if the CCS codes described in Section 4.2 are recorded within 10 days before starting CRRT, which reflects the optimal window of the comprehensive model.

		UCLA CRRT			Cedars Sinai CRRT			UCLA Control
		Overall	Do not recommend	Recommend	Overall	Do not recommend	Recommend	Do not recommend
n		4161	1920	2241	3263	1462	1801	4725
Outcome, n (%)								
	Comfort Care	1179 (28.3)	1179 (61.4)	-	984 (30.2)	984 (67.3)	-	-
	Expired	741 (17.8)	741 (38.6)	-	478 (14.6)	478 (32.7)	-	-
	Recovered renal function	602 (14.5)	-	602 (26.9)	704 (21.6)	-	704 (39.1)	-
	Transitioned to hemodialysis	1639 (39.4)	-	1639 (73.1)	1097 (33.6)	-	1097 (60.9)	-
Total days on CRRT, median [Q1,Q3]		6.0 [3.0,12.0]	5.0 [2.0,12.0]	6.0 [3.0,12.0]	5.0 [3.0,10.0]	4.0 [2.0,9.0]	6.0 [4.0,11.0]	0.0 [0.0,0.0]
Year of CRRT, median [min,max]		2018 [2015,2021]	2018 [2015,2021]	2018 [2015,2021]	2019 [2016,2021]	2019 [2016,2021]	2019 [2016,2021]	2018 [2005,2022]
Number of previous CRRT Treatments, mean (SD)		0.2 (0.7)	0.1 (0.5)	0.3 (0.8)	0.2 (0.5)	0.1 (0.4)	0.2 (0.5)	0.0 (0.0)
Age, median [Q1,Q3]		60.0 [47.0,69.0]	60.0 [48.0,69.0]	59.0 [45.0,68.0]	66.0 [56.0,75.0]	68.0 [58.0,78.0]	63.0 [53.0,73.0]	61.0 [49.0,70.0]
Sex, n (%)								
	Female	1682 (40.4)	765 (39.8)	917 (40.9)	1111 (36.3)	537 (37.8)	574 (35.0)	1877 (39.7)
	Male	2479 (59.6)	1155 (60.2)	1324 (59.1)	1946 (63.7)	882 (62.2)	1064 (65.0)	2848 (60.3)
Ethnicity, n (%)								
	Hispanic or Latino	1416 (34.0)	580 (30.2)	836 (37.3)	756 (24.7)	307 (21.6)	449 (27.4)	1548 (32.8)
	Not Hispanic or Latino	2745 (66.0)	1340 (69.8)	1405 (62.7)	2301 (75.3)	1112 (78.4)	1189 (72.6)	3177 (67.2)
Race, n (%)								
	American Indian or Alaska Native	15 (0.4)	11 (0.6)	4 (0.2)	4 (0.1)	1 (0.1)	3 (0.2)	70 (1.5)
	Asian	337 (8.1)	163 (8.5)	174 (7.8)	249 (8.1)	103 (7.3)	146 (8.9)	386 (8.2)
	Black or African American	429 (10.3)	199 (10.4)	230 (10.3)	462 (15.1)	226 (15.9)	236 (14.4)	494 (10.5)
	Multiple Races	42 (1.0)	15 (0.8)	27 (1.2)	115 (3.8)	40 (2.8)	75 (4.6)	58 (1.2)
	Native Hawaiian or Other Pacific Islander	21 (0.5)	10 (0.5)	11 (0.5)	12 (0.4)	3 (0.2)	9 (0.5)	22 (0.5)
	Unknown	1268 (30.5)	555 (28.9)	713 (31.8)	500 (16.4)	287 (20.2)	213 (13.0)	1499 (31.7)
	White or Caucasian	2049 (49.2)	967 (50.4)	1082 (48.3)	1715 (56.1)	759 (53.5)	956 (58.4)	2196 (46.5)
Heart comorbidities, n (%)								
	No	1798 (43.2)	839 (43.7)	959 (42.8)	735 (22.5)	246 (16.8)	489 (27.2)	3319 (70.2)
	Yes	2363 (56.8)	1081 (56.3)	1282 (57.2)	2528 (77.5)	1216 (83.2)	1312 (72.8)	1406 (29.8)
Liver comorbidities, n (%)								
	No	2271 (54.6)	1046 (54.5)	1225 (54.7)	1536 (47.1)	605 (41.4)	931 (51.7)	3624 (76.7)
	Yes	1890 (45.4)	874 (45.5)	1016 (45.3)	1727 (52.9)	857 (58.6)	870 (48.3)	1101 (23.3)
Infection comorbidities, n (%)								
	No	1878 (45.1)	845 (44.0)	1033 (46.1)	1121 (34.4)	376 (25.7)	745 (41.4)	3648 (77.2)
	Yes	2283 (54.9)	1075 (56.0)	1208 (53.9)	2142 (65.6)	1086 (74.3)	1056 (58.6)	1077 (22.8)

**Table A2:** Optimal parameters (model, window, and feature selection correlation threshold) and number of features before and after training for all experiments. Simple imputation and feature selection with a correlation threshold were optimal for all experiments. Note that for each experiment, the feature counts correspond to the features available at the optimal window after hyperparameter tuning. The feature counts after training also correspond to the optimal feature selection method after hyperparameter tuning.

				Number of features before training		Number of features after training	
	Model	Window (days)	Correlation threshold	Raw features	Total features (engineered)	Raw features	Total features (engineered)
UCLA Model	xgb	5	0.08	2350	8974	131	187
Cedars Sinai Model	lgb	6	0.05	1050	2989	212	281
UCLA + Cedars Sinai Model	lgb	10	0.02	3044	12379	1338	2838
UCLA + Cedars Sinai + Control Model	xgb	7	0.04	3238	13321	289	354
UCLA Model, Feature Intersection of All Cohorts	xgb	5	0.08	575	1024	61	80
UCLA Model, Feature Intersection of UCLA and Cedars Sinai	xgb	5	0.08	651	1121	60	80
Cedars Sinai Model, Feature Intersection of All Cohorts	lgb	6	0.05	595	1052	128	162
Cedars Sinai, Feature Intersection of UCLA and Cedars Sinai	xgb	14	0.035	703	1240	268	384
UCLA + Cedars Sinai Model, Feature Intersection of All Cohorts	lgb	10	0.02	635	1131	377	638
UCLA + Cedars Sinai Model, Feature Intersection of UCLA and Cedars Sinai	lgb	10	0.02	696	1218	411	687
UCLA + Cedars Sinai + Control Model, Feature Intersection of All Cohorts	lgb	10	0.01	635	1131	456	778
